# Automated word class and person usage metrics as markers of Alzheimer’s and frontotemporal dementia

**DOI:** 10.1002/alz.085437

**Published:** 2025-01-09

**Authors:** Pamela Lopes da Cunha, Fabian Ruiz, Franco Javier Ferrante, Lucas Sterpin, Agustin Ibañez, Andrea Slachevsky, Diana Matallana, Angela Martinez, Eugenia Hesse, Adolfo M Garcia

**Affiliations:** ^1^ Cognitive Neuroscience Centre, University of San Andres, Victoria, Buenos Aires Argentina; ^2^ Cognitive Neuroscience Center, University of San Andrés, Victoria, Buenos Aires Argentina; ^3^ Global Brain Health Institute (GBHI), Trinity College Dublin (TCD), Dublin, Dublin Ireland; ^4^ Latin American Brain Health (BrainLat) Institute, Universidad Adolfo Ibáñez, Peñalolén, Santiago Chile; ^5^ Servicio de Neurología, Departamento de Medicina, Clínica Alemana‐Universidad del Desarrollo, Santiago Chile; ^6^ Pontificia Universidad Javeriana, Bogota, Cundinamarca Colombia; ^7^ School of Medicine and Health Sciences, University of Rosario, Bogota, Bogota Colombia; ^8^ Global Brain Health Institute, University of California, San Francisco, CA USA

## Abstract

**Background:**

Dementia impacts the way individuals perceive and describe everyday events. Alzheimer's disease (AD) notably affects processing of entities manifested by nouns, while behavioral variant frontotemporal dementia (bvFTD) often presents a detached, third‐person perspective. Yet, the potential of natural language processing tools (NLP) to detect these variations in spontaneous speech remains explored. To tackle this gap, we analyzed both patterns via automated discourse‐level metrics in individuals with AD and bvFTD, contrasting them with healthy controls (HCs).

**Methods:**

Persons with AD (n = 21), bvFTD (n = 21), as well as HCs (n = 21), narrated a typical day of their lives. We analyzed the frequency of nouns and verbs, along with first‐ or third‐person usage, via part‐of‐speech and morphological tagging, respectively. Inferential statistics and machine learning were used to examine whether these features were useful for discriminating patients from HCs at both the group and the subject level. We further evaluated whether such features correlated with cognitive symptom severity, as captured through the Montreal Cognitive Assessment (MoCA).

**Results:**

Compared with HCs, AD (but not bvFTD) patients exhibited a lower proportion of nouns, without differences in verb ratio. Conversely, persons with bvFTD (but not those with AD) had a greater proportion of third‐person markers and a reduced proportion of first‐person markers. Machine learning analyses showed that these features robustly identified individuals within each group (AUCs = 0.75). No linguistic feature was significantly correlated with MoCA scores in either patient group.

**Conclusions:**

Spontaneous daily narratives offer distinct markers for AD and bvFTD, detectable through automated analysis. Focusing on specific linguistic attributes relevant to each type of dementia not only aids in understanding but also enhances diagnosis and tracking of these conditions. Overall, our findings attest to the relevance of NLP tools as a viable, cost‐effective means to identify scalable dementia markers.

